# Embedding, aligning and reconstructing clinical notes to explore sepsis

**DOI:** 10.1186/s13104-021-05529-4

**Published:** 2021-04-14

**Authors:** Xudong Zhu, Joseph M. Plasek, Chunlei Tang, Wasim Al-Assad, Zhikun Zhang, Yun Xiong, Liqin Wang, Sharmitha Yerneni, Carlos Ortega, Min-Jeoung Kang, Li Zhou, David W. Bates, Patricia C. Dykes

**Affiliations:** 1 Shanghai Key Laboratory of Data Science, School of Computer Science, Fudan University, Shanghai, China; 2 Division of General Internal Medicine and Primary Care, Brigham and Women’s Hospital, Harvard Medical School, Boston, MA USA; 3 Clinical and Quality Analysis, Mass General Brigham, Boston, MA USA; 4 College of Nursing, The Catholic University of Korea, 222 Banpo-daero, Seocho-gu, Seoul, 06591 South Korea

**Keywords:** Sepsis, Representation learning, Exploratory analysis, Data driven medicine

## Abstract

**Objective:**

Our goal was to research and develop exploratory analysis tools for clinical notes, which now are underrepresented to limit the diversity of data insights on medically relevant applications.

**Results:**

We characterize how exploratory analysis can affect representation learning on clinical narratives and present several self-developed tools to explore sepsis. Our experiments focus on patients with sepsis in the MIMIC-III Clinical Database or in our institution’s research patient data repository. We found that global embeddings assist in learning local representations of clinical notes. Second, aligning at any specific time facilitates the use of learning models by pooling more available clinical notes to form a training set. Furthermore, reconstruction of the timeline enhances downstream-processing techniques by emphasizing temporal expressions and temporal relationships in clinical documentation. We demonstrate that clustering helps plot various types of clinical notes against a scale, which conveys a sense of the range or spread of the data and is useful for understanding data correlations. Appropriate exploratory analysis tools provide keen insights into preprocessing clinical notes, thereby further enhancing downstream analysis capabilities, making data driven medicine possible. Our examples can help generate better data representation of clinical documentation for models with improved performance and interpretability.

**Supplementary Information:**

The online version contains supplementary material available at 10.1186/s13104-021-05529-4.

## Introduction

Sepsis, a global health concern [1], is defined as “life-threatening organ dysfunction caused by a dysregulated host response to infection [2, 3].” With high rates of morbidity, readmission, and mortality, [3–6], sepsis is considered one of the 12 leading causes of death in the United States [7]. Although previous work highlighted that sepsis has a vicious cycle in which inflammation induces and exacerbates coagulopathies and organ damage [8, 9], the precise description of each sepsis episode (e.g., duration, pattern) remains unclear. Further, there is very limited data on the clinical relevance and impact of some pathogens, (e.g., anaerobic bacteria) in sepsis [2].

Data-driven medicine has not only the potential to improve the speed and accuracy of diagnosis but to unlock the possibility of personalized medical treatments. However, the underrepresentation of exploratory analysis tools for clinical notes has limited the diversity of data insights on medically relevant applications. Exploratory analysis, which goes beyond basic initial data analysis tasks (i.e., sort, filter, aggregate, correlate, group, derive attributes), assists in gaining insights from raw data prior to training learning models [10]. Clinical notes can contain summaries (e.g., the history of present illness section) that describe and illustrate the longitudinal course of particular clinical events or situations experienced by patients [11, 12]. There are obstacles to machine understanding capabilities due to the large amount of information recorded in clinical notes. These challenges arise as clinical notes may follow specific formats (e.g., templates) and may contain redundancies, misspellings, relationships, negations, and abbreviations that affect the clinical representation of concepts. Researchers have framed these various note-related challenges as deep or machine learning tasks and have adopted different algorithms to tackle them. A common strategy is to transform the notes into an appropriate data representation for downstream analysis. Nevertheless, learning to generate better representations may involve a hierarchy of representations from object parts to scenes, which require different levels of granularity. The lack of exploratory analysis tools with appropriate data preprocessing abilities has restricted models to those exhibiting poor performance and interpretability.

In this study, we characterize how exploratory analysis can affect representation learning on clinical narratives and present several self-developed tools to explore sepsis.

## Main text

### Methods

#### Embeddings

Embeddings have dominated the proceedings of conferences in recent years; for example, word embeddings (e.g., word2vec [13]) can be generated using various methods such as neural networks, co-occurrence matrix, and probabilistic models. It should be noted that embeddings as a lower-dimensional representation of data can offer both global (e.g., sentence or document level embeddings) and local (e.g., word embeddings) perspectives. A word embedding typically utilizes the bag-of-words model, a standard choice in representation learning, combined with substantial preprocessing [14]. For example, the bag of words with TF-IDF weighting representation dominates others with larger sample size [15]. Roberts et al. [16] used a comprehensive set of features in his classification of semantic relations: context features (e.g., n-gram), nested relation features (connections in the text span between candidate pairs of concepts), single concept features (e.g., covered words and concept types), Wikipedia features (e.g., concepts matching Wikipedia titles), concept bi-grams features, and similarity features. However, the bag of words model is inherited from the implicit one-hot encoding of words. One main approach to overcome the defects is to use explicit domain knowledge, namely expert-curated techniques developed in natural language processing (NLP) applications. Topic models such as latent Dirichlet allocation (LDA) [17] represent another alternative; however, aggregation for the LDA representation significantly underperforms the bag of words representation except when using very small training sets.

Embeddings can also be used to compute the similarity in meaning between short and long text. We implemented an algorithm based on Charikar’s SimHash [18] under the *K*-means clustering paradigm to help with local representation learning [19]. Our algorithm (1) embedded each clinical document into a fingerprint, (2) partitioned those fingerprints into several (e.g., *K* = 10) clusters, (3) designated each cluster representative as an example, which is a fingerprint of real clinical notes closest to the centroid, and then (4) learned the local representation from the examples (e.g., feature engineering, labeling). Document embedding can be replaced by representative text segments (i.e., sections of the clinical document).

#### Data alignment

**Figure 1:**
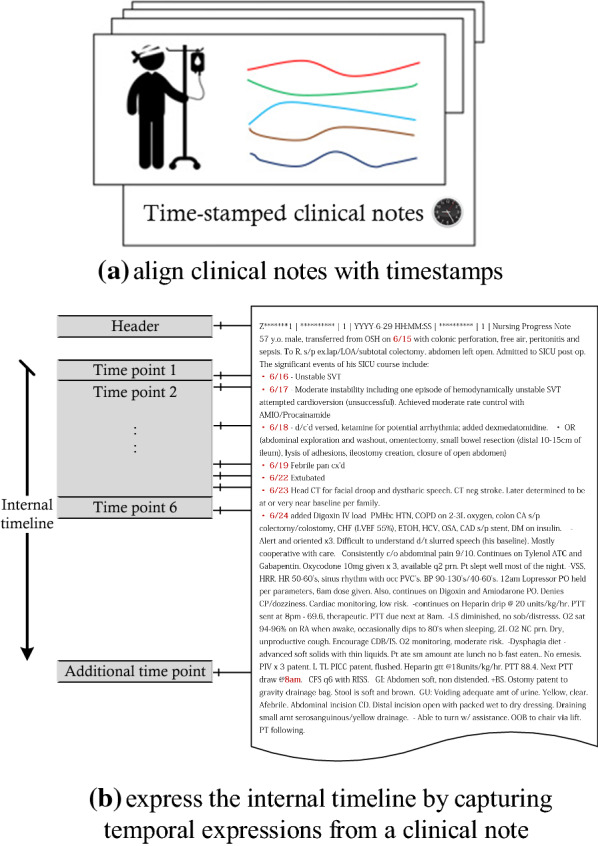
An illustration of the timelines of clinical notes

Alignment allows data to fetched efficiently. As shown in Fig. 1, there exist multiple timelines (i.e., at least two) in a clinical corpus. The external timeline (see Fig. 1b) can be arranged at the level of either a patient or a clinical note. It is easy to understand how to insert notes from each domain into the appropriate chronological place for the patients in the corpus. Our previous work [20, 21] aligned the clinical corpus (based on patient death time or discharge time) in chronological order at the level of the clinical notes, which can provide a larger training set for deep or machine learning models.

Alternatively, alignment can be annotated based on temporal expressions in the document. The internal timeline (see Fig. 1a) is located in a clinical document by capturing temporal expressions. Temporal expressions found within these notes provide cues about relationships between clinical events. While useful for subsequent analysis tasks, learning temporal expressions is challenging due to the variety of ways in which they are expressed, as they can be based on a start time (e.g., a medication administration), qualitative constraint representation (e.g., days prior to death), or duration-based representation (e.g., a sepsis episode, a hospital stay) [22]. One common practice to obtain temporal expressions is to retrieve the temporal dimension of existing objects (i.e., the creation time for a specific clinical entity) and utilize this as a temporal component. Another approach utilizes TimeML to annotate all time-oriented information of task-specific entries (http://www.timeml.org) [23] or other markup languages to meet the requirements of temporal reasoning tasks. However, both practices have shortcomings: while the former is too naïve to effectively process detailed information on clinical entities, the latter relies on rule-based natural language processing capabilities that require manual effort to recognize novel temporal patterns. Jia et al. [24] suggest annotating any temporal expression in a clinical document that contains at least one of the following: (a) explicit time expressions (e.g., dates, times); (b) implicit temporal signals (i.e., cue words for temporal relations); (c) ordinal words (e.g., ‘first’).

#### Data reconstruction

Data reconstruction is mostly used to process time series data to solve the information loss issue. We executed a classic pattern mining PrefixSpan [25] by considering a complete set of ordered frequent patterns.

We developed a data reconstruction algorithm [26] to transform free-text clinical notes into a set of time-stamped (or time-anchored) clinical entities, which happen to be represented in a sequential data format. First, the data reconstruction algorithm detects if an expression has temporal intent. Second, it decomposes and rewrites the expression into non-temporal sub-expressions and temporal constraints. Finally, it clusters similar non-temporal sub-expressions by using an unsupervised sentence embedding under the modified K-medoids paradigm. Consider a sequential dataset of sepsis symptoms (e.g., fever, hypothermia, tachycardia) that includes patterns such as “fever reaches peak before a sharp drop in blood pressure.” A time expression can also be associated with each attribute. For example, each record could be the sepsis history of a patient, with a listing of clinical entities recorded at different times. Using the temporal information, it is possible to detect patterns such as “patients who are sepsis survivors tend to experience sepsis recurrence in the period immediately following hospital discharge.” Additional file 1: Table S1 shows an example of sequential data: there are five different times—*t*_*1*_, *t*_*2*_, *t*_*3*_, *t*_*4*_, *t*_*5*_; three different patients—*P*_*1*_, *P*_*2*_, *P*_*3*_, and five different sepsis symptoms—*A*, *B*, *C*, *D*, *E*. In the top half of Additional file 1: Table S1, each row corresponds to the symptoms recorded at a particular time for each patient: e.g., at time *t*_*3*_, patient *P*_*2*_ had symptoms *A* and *D*. In the bottom half of Additional file 1: Table S1, ordering is instead by patient: e.g., patient *P*_*3*_ experienced symptoms *A* and *C* at time *t*_*2*_.

### Results

Our embedding-based exploratory analysis tool can assist in a variety of informatics related tasks with an *O*(*n*) time complexity. These tasks include the detection of clinical sublanguages and the automated generation of prototype templates.

As shown in Additional file 1: Table S2, we merged sepsis nursing notes related to the same patient with adjacent time periods together. The format for reconstruction results in sequential data that includes information on the “cause of sepsis,” “symptoms related to sepsis”, and “duration (days or hours) between clinical entities (e.g., symptoms).” The underlined sentence in Additional file 1: Table S2 is our target. Based on data alignment and reconstruction, two records were generated corresponding to Additional file 1: Table S2′s highlighted part:

- Non-Hodgkin’s lymphoma (caused sepsis): fever, 2 days or 42 h (i.e., from DD-MM-YYYY 10:02 to + 2 DD-MM-YYYY 04:26)
- Non-Hodgkin’s lymphoma: hypotension, 2 days or 42 h

The possible downstream analysis of reconstruction results may include risk prediction or pattern mining. Making predictions with sequences occurs in a variety of ways. A commonly used method involves predicting the next value for a given input sequence. For instance, framing the problem as “does fever occur in this case of sepsis within a specified time?” is a sequence classification task that involves predicting a class label for a given input sequence. Given the clinical entity “fever:” for example, it is easy to use sequential data to estimate: (1) whether fever “occurred” or “did not occur” during an episode of sepsis, (2) the duration of the “fever,” and (3) the relationship between the “fever” and other clinical entities (e.g., tachycardia, tachypnoea, blood leukocyte changes).

We found a total of 957 patterns from PrefixSpan pattern mining of all corpora (see Table 1 with PR_Dc). We obtained similar and comparable results on the public MIMIC-III Clinical Database [27] as shown in Table 1.

**Table 1:** The top 10 results of sepsis symptom patterns compared in the private and public datasets

Corpus	Freq	Pattern
PR_Dc	76	[‘respiratory failure’, ‘hypoxemic respiratory’, ‘hypoxemic resp’]
	23	[‘respiratory failure’, ‘acute hypoxemic’, ‘hypoxemic respiratory’, ‘hypoxemic resp’]
	16	[‘respiratory failure’, ‘worsening respiratory’, ‘respiratory status’]
	14	[‘cystic lesion’, ‘6 cm cystic’, ‘septated cystic’, ‘abscess drainage’, ‘felt SOB’]
	13	[‘respiratory distress’, ‘purulent drainage’, ‘denies chill’]
	13	[‘LLE cellulitis’, ‘redness noted’]
	12	[‘hypoxemic respiratory’, ‘developed hypoxemic’, ‘hypoxemic resp’]
	11	[‘echinococcal cysts’, ‘showing cystic’, ‘cystic lesion’]
	10	[‘respiratory failure’, ‘respiratory distress’, ‘acute respiratory’]
	8	[‘hepaitic lesion’, ‘cystic lesion’, ‘septated cystic’, ‘abscess drainage’, ‘felt SOB’]
MIMIC-III	34	['altered mental’, 'hypercarbic respiratory']
	22	['yellow secretion’, 'respiratory failure']
	18	['respiratory failure’, 'breathing noted']
	17	['respiratory distress’, 'white secretions']
	9	['breath sounds', 'tan secretions']
	8	['respiratory failure’, 'thick secretion']
	6	['breath sounds', 'thick secretion']
	5	[‘cystitis', 'secretions suctioned’, 'mouthing words’]
	4	['tinged secretions’, ’uncomfortable']
	2	[‘abdominal discomfort’, ‘brown drainage’, ‘hypercarbic respiratory’, ‘pulm edema’]

### Discussion and conclusions

Our main finding was that it is possible to develop novel exploratory analysis tools to improve representation learning on clinical narratives to explore sepsis. The ability for exploratory analysis tools to embody scalability and usability features conveys detailed information related to clinical disease progression, which could be applied to inform therapeutic and disease management decisions. Appropriate exploratory analysis tools provide a keen insight into clinical notes to help generate better data representations for models with improved performance and interpretability. For example, although deidentified open access data lack available time expressions, we obtained similar and comparable pattern results with our private corpora by only considering the sequence in time.

We found that global embeddings assist in learning local representations of clinical notes. Data alignment at any specific time facilitates the use of learning models by increasing the size of the training set. Reconstruction of the data enhances downstream-processing techniques by emphasizing useful representations (e.g., temporal expression) in clinical documentation. We demonstrated how clustering can help plot various types of clinical notes against a scale, which conveys a sense of range or spread of the data and is useful in understanding data correlations.

As our study investigated methods for exploratory analysis of general clinical notes corpora instead of patients who are carefully chosen from clinical trials, our findings provide new insights into sepsis using real world data. This approach simplifies the process of knowledge abstraction from clinical practice for practical applications in clinical research.

## Limitations

One limitation in our study is that we only used data distribution to explore the corpus. While nursing and physician progress and discharge summaries for a patient may have various correlations to different stages of sepsis progression, merging them to apply learning methods to compute a score to balance the differences (i.e., priority, dataset size) among the clinical documents may not be ideal. For example, we did not consider the potentially complex relationships among the corpora nor any structured clinical data (i.e., symptoms documented in the patients’ problem list in the EHR).

## Supplementary Information

**Additional file 1: Table S1.** Understanding sequence data. **Table S2.** An example of data alignment at the patient level. **Table S3.** The list of headers for all described clinical notes.

## Data Availability

Mass General Brigham research data (i.e., the two corpus of clinical notes from our institutions research patient data repository) are unavailable for access because they are confidential, and it would be cost prohibitive to sufficiently de-identify such a large corpus of clinical documents to remove all patient identifying data according to the HIPAA standard. A. Private sepsis corpora We gathered critical care nursing notes, physician progress notes, and discharge summaries from our research patient data registry at Mass General Brigham, a large integrated healthcare delivery network located in Boston, Massachusetts. Since the proportion of patients with sepsis is lower than that of other diseases, we extracted data twice to construct two corpora. Each extracted document contains a header listing information related to the clinical notes (see Additional file 1: Table S3). 1) PR_Nn: a private sepsis corpus containing critical care nursing notes Nursing notes: We retrieved 10,713 free-text nursing progress notes containing the keyword “sepsis” corresponding to 1351 unique patients between 2015 and 2017. The maximum length of notes was 200 words. By comparing NoteID and LineNBR to filter repeated notes of the same patients’ to find the most recent note for the nursing encounter, we obtained a total of 4877 unique nursing notes. The average number of nursing notes per patient was 2.68, with a maximum of 51 and a minimum of 1. 2) PR_Dc: a private sepsis corpus extracted from diabetic cases Physician progress notes: We retrieved 1872 unique patients among 459,280 cases with diabetes diagnosis seen between July 1st, 2017 and June 30th, 2018, whose “Principle Diagnosis” or “Diagnosis *n* (*n* = [1, 2, …, 10])” showed an ICD-10 code related to sepsis/septic shock (R65.20/R65.21).There was a total of 100,331 free-text physician progress notes corresponding to 1210 unique providers between July 1st, 2017 and June 30th, 2018. The maximum length of physician progression notes was 7500 words. Overall, 5 format types including significant event, emergency department observation, learning and development delivery, perioperative nursing, and lactation notes, both system pre-defined types and clinicians’ free-text entries, were found in this type of clinical notes. Approximately two-thirds of the documents used system pre-defined types. Authorship was unevenly distributed, with a small proportion of the clinicians responsible for a large proportion of notes. The average number of physician progress notes per patient per day in one hospital stay was 1.05 notes. Discharge summaries: We retrieved 3573 free-text discharge summaries corresponding to the same hospital stays/patients described in the physician progress notes section above. The maximum length of discharge summaries was 4000 words. The average duration for a hospital stay was 9.80 days, with a maximum duration of 15 days and a minimum duration of 1 day. The average number of discharge summaries per patient was 4.97 documents, indicating that most sepsis patients were hospitalized multiple times. B. Public sepsis corpora: MIMIC_III MIMIC-III [27], run by the MIT PhysioNet Team, integrates deidentified, critical care clinical data of patients admitted to the Beth Israel Deaconess Medical Center in Boston, Massachusetts between 2001 and 2012. The open nature of the data allows clinical studies to be reproduced and improved in ways that would not otherwise be possible. Based on an explicit ICD-9-CM code (i.e., 995.92 for severe sepsis), we extracted 11,335 clinical notes from 1157 unique patients. Relevant note types include, but not are limited to, generic, intensivist, and ICU event notes. The average number of notes per patient was 6.02.

## Abbreviations

EHR: Electronic health record
IRB: Institutional review board
NLP: Natural language processing
ICD-10-CM: International Classification of Diseases—Version 10

## Code availability

Project home page: https://github.com/zzky/tech_note.

Operating system(s): Platform independent.

Programming language: Python.
