# Supplementary material for: Embedding, aligning and reconstructing clinical notes to explore sepsis
Source: BMC Res Notes. 2021 Apr 14;14:136. doi: 10.1186/s13104-021-05529-4 (PMC8048212; doi:10.1186/s13104-021-05529-4)
Supplement: Supplementary file 1 — Additional file 1: Table S1. Understanding sequence data. Table S2. An example of data alignment at the patient level. Table S3. The list of headers for all described clinical notes. [file 13104_2021_5529_MOESM1_ESM.docx]

# Additional Material

Table S1. Understanding sequence data.

Table S2. An example of data alignment at the patient level.

Table S3. The list of headers for all described clinical notes.

1. Understanding Sequence Data

| TID | Sepsis Symptoms |
| --- | --- |
| *t_1_* | *P_1_: A, B* |
| *t_2_* | *P_3_: A, C* |
| *t_2_* | *P_1_: C, D* |
| *t_3_* | *P_2_: A, D* |
| *t_4_* | *P_2_: E* |
| *t_5_* | *P_1_: A, E* |
| *P_1_* | *(t_1_: A, B), (t_2_: C, D), (t_5_: A, E)* |
| *P_2_* | *(t_3_: A, D), (t_4_: E)* |
| *P_3_* | *(t_2_: A, D)* |

(*t*: time point; *P*: patient; *A-E*: symptoms)

1. An Example of Data Alignment at the patient Level

| Time | PR_Nn NoteTXT |
| --- | --- |
| *DD-MM-YYYY 10:02* | Patient admitted with sepsis, BMT years ago with GVHD, transferred from CCH. Blood cultures positive, being treated for sepsis as workup continues. Pt lives in [LOCATION] with his wife and children. He was independent prior to this episode. No services on admission. … Will follow with team for care transitions planning. |
| +2 DD-MM-YYYY 04:26 | 66 y.o. male with history of Non-Hodgkin’s lymphoma s/p BMT ([DATE]) w/ h/o GVHD (chronic steroids), DM (on insulin), p/t OSH with hypotension and leukocytosis to 18, transferred to [HOSPITAL] ICU for management of sepsis, treated with IVF, stress dose steroid,ids, broad-spectrum antibiotics, now with strep viridans bacteremia and resolved sepsis on Ceftriaxone. Transferred to BMT-B on [DATE]. Admits 6A from 2 day stay in 3B w/ co fevers, hypotension, s/p echo, corrected with hydration and abx therapy, now independent w/ VSS, Afeb, MAE, independent, abrasion to LLE OTA, on AC & HS CZI, and consistent carb/BMT diet ad lib. Vital signs stable, afebrile, drinking and eating, no complaints of pain. Continue supportive care. Probable discharge mid week. |
| +2 DD-MM-YYYY 16:10 | [PATIENT NAME] was admitted to [HOSPITAL] on [DATE] with NHL s/p BMT in [date] with sepsis. Discussed pt with team and pt will be ready for discharge tomorrow or [date] pending PICC line placement. Met with pt to review plan and collaborate regarding referrals. Pt is in agreement to discharge home [LOCATION] with IV antibiotics and PICC line care. |

1. The List of Headers for All Described Clinical Notes

| Corpus | Header |
| --- | --- |
| PR_Nn | PATIENT_NUM\|ENCOUNTER_NUM\|NOTETYPE\|CREATELOCALDTS\|NOTEID\|LINENBR\|NOTETXT |
| PR_Dc | EMPI\|EPIC_PMRN\|MRN_TYPE\|MRN\|REPORT_NUMBER\|REPORT_DATE_TIME\|REPORT_DESCRIPTION\|REPORT_STATUS\|REPORT_TYPE\|REPORT_TEXT |
| MIMIC_III | HADM_ID\|ICD9_CODE\|NOTE_TEXT\|PATIENT_ID\|CHARTDATE\|CHARTTIME\|SUBJECT_ID |
